# Earliest depiction of port wine stains, eye signs and ear deformity in the portrait of Edward Grimston (c. 1399–1478) by Petrus Christus

**DOI:** 10.1007/s00415-025-13575-2

**Published:** 2025-12-22

**Authors:** Hutan Ashrafian, Leanne Harling

**Affiliations:** 1 The Department of Surgery and Cancer, Institute of Global Health Innovation, Imperial College London, St Mary’s Hospital, 10th Floor Queen Elizabeth the Queen Mother (QEQM) Building, Praed Street, London, W2 1NY UK; 2 Department of Surgical Oncology, School of Cancer and Pharmaceutical Sciences, King’s College London, Guys Hospital, 6th Floor, Borough Wing, Great Maze Pond, SE1 9RT UK

**Fig. 1:**
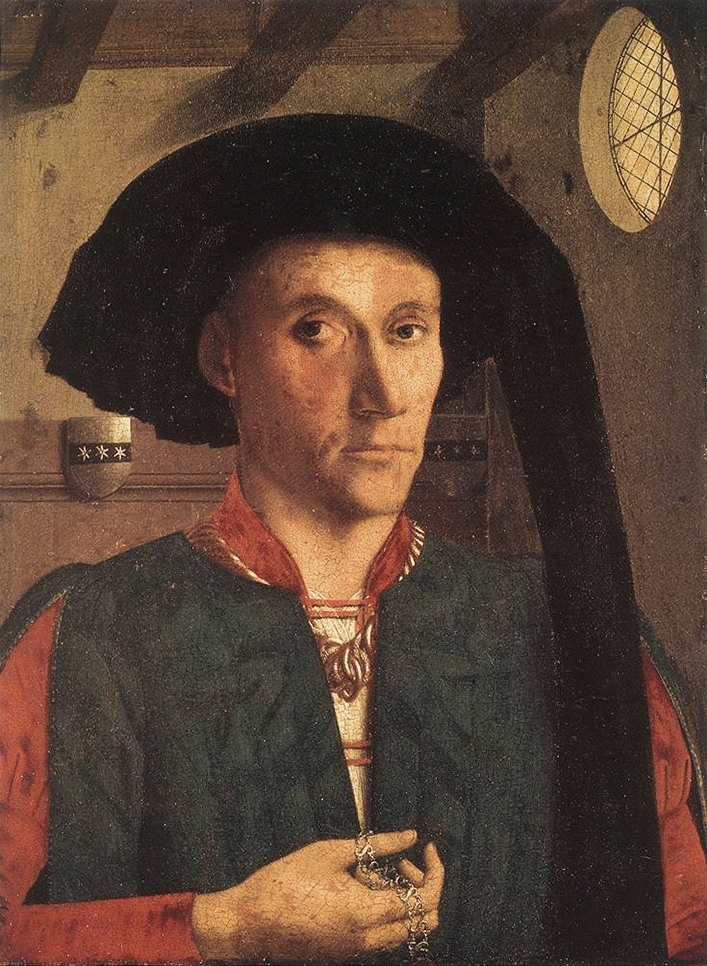
Portrait of Edward Grimston (c. 1446) by Petrus Christus, © The National Gallery, London (The image reproduced in this manuscript is in the public domain and may be freely used without restriction)

Edward Grimston (c. 1399–1478) was an English court official active under King Henry VI, noted for his diplomatic and administrative roles. He served on missions to the Burgundian court, where he likely commissioned his portrait (Fig. 1) from the Netherlandish painter Petrus Christus. This portrait, dated to 1446, is now held in the National Gallery, London, and is among the early examples of independent portraiture in Northern Europe. Grimston’s activities reflect the broader political and cultural exchanges between England and Burgundy in the mid-fifteenth century.

Contemporary historical documentation suggests Grimston maintained sufficient health and stamina to fulfill demanding diplomatic duties in various courts, implying no severe congenital impairment. Nonetheless, his portrait by Petrus Christus (Fig. 1) reveals four salient clinical features: (1) port-wine stains (PWS) localized to the ophthalmic (V1) and maxillary (V2) trigeminal dermatomes; (2) deformity of the external ear (auricle), with a malformed helix, possible accessory auricular remnant, and absent lobule; (3) strabismus characterized by right hypertropia with exotropia; and (4) possible left hemifacial weakness (as the right side seems to present facial muscle activity). While formal examination of these findings is limited by the retrospective nature of portrait analysis, they remain clinically suggestive of an underlying neurocutaneous or craniofacial syndrome.

The presence of a PWS in the V1–V2 distributions aligns with Sturge–Weber syndrome in an adult, a phakomatosis most commonly associated with a specific somatic mosaic mutation in the *GNAQ* gene and characterized by capillary malformations, leptomeningeal angiomas, seizures which are typically present in 83% of adult individuals, neurological deficit in 65% and ocular involvement, such as glaucoma present in approximately 60% of adults with this syndrome [1–3]. Although the classic triad may not be fully documented here, an incomplete or partial manifestation can present with a facial PWS and neurological or ocular findings. The right ear deformity is not characteristic of typical Sturge–Weber, but complex congenital anomalies can occur in less typical variants. The left hemifacial weakness and ocular deviation may reflect cranial nerve involvement or subtle hemispheric dysfunction associated with the syndrome.

Other differential diagnoses here could include Neurofibromatosis Type 1 (NF1): typically characterized by café-au-lait spots (possible in this portrait), neurofibromas, and Lisch nodules, which are absent in this depiction. Alternatively, PHACE syndrome (posterior fossa malformations, hemangiomas, arterial lesions, cardiac anomalies, eye abnormalities) often presents with large facial hemangiomas rather than port-wine stains, making it less compatible with Grimston’s findings. Tuberous sclerosis, the features of which commonly include facial angiofibromas rather than port-wine stains, along with other hallmark findings (e.g., cortical tubers, periungual fibromas). Oculo-auriculo-vertebral spectrum (e.g., Goldenhar syndrome) may present with ear anomalies and facial asymmetry, but the presence of a segmental PWS and specific trigeminal distribution is not typical. Congenital facial nerve palsies or isolated craniofacial malformations could explain selective ear and facial anomalies, but would not sufficiently account for the port-wine stain in the trigeminal region.

Despite these physical findings and the image representing the earliest depiction of a port-wine stain, Grimston’s documented diplomatic engagements imply these conditions did not preclude him from contributing effectively to 15th-century society. Moreover, the meticulous attention to anatomical detail by Netherlandish painters such as Petrus Christus provides a rare clinical window into past figures, enabling modern analyses of possible medical conditions centuries later.
